# Voice Disorders Among Teachers in Taif City, Kingdom of Saudi Arabia

**DOI:** 10.7759/cureus.54561

**Published:** 2024-02-20

**Authors:** Yahya A Fageeh, Turki A Alotaibi, Nawaf Saleh A Althobaiti, Alhanouf A Alkhaldi, Abdullah A Althobaiti, Hanouf A Althobaiti, Liyan K Abu Rukbah, Shuruq A Alharati, Marwan F Alnofaie

**Affiliations:** 1 Otorhinolaryngology - Surgery, College of Medicine, Taif University, Taif, SAU; 2 Otorhinolaryngology, King Faisal Medical Complex, Taif, SAU

**Keywords:** voice-related symptoms, hoarseness, associated factors, voice handicap index, occupational factors, observational study, demographic characteristics, cross-sectional, teaching-related, vocal health

## Abstract

Background

Voice disorders (VD) pose significant challenges for teachers as they impact their professional and personal lives. Teaching requires extensive use of the voice, making teachers particularly susceptible to vocal health issues. VD can hinder the quality of education.

Objectives

This study aimed to comprehensively explore the prevalence, associated factors, and impact of VD among teachers and their health-seeking behavior regarding VD.

Methodology

A cross-sectional observational study was conducted in Taif City, Kingdom of Saudi Arabia (KSA), between November and December 2023. The data were collected through a questionnaire covering demographic variables, occupational factors, voice-related symptoms, associated health conditions, and the impact of VD. The statistical analysis was performed using SPSS Statistics version 26 (IBM Corp. Released 2019. IBM SPSS Statistics for Windows, Version 26.0. Armonk, NY: IBM Corp.), and chi-squared tests were used to assess associations.

Results

The study involved 568 teachers, 61.8% of whom had VD. The study identified significant associations between VD and demographic characteristics, habits, and teaching-related variables. Age, gender, teaching subject, class size, and weekly teaching load were associated with the prevalence of VD. Hoarseness, throat pain, and throat dryness were prevalent symptoms among teachers with VD. The impact of VD on teachers was evident, with a considerable proportion reporting work absenteeism (28.7%) and even contemplating retirement due to voice problems (6.3%).

Conclusion

This study offers a nuanced understanding of VD among teachers in the Taif region, emphasizing the complex interplay of demographic, symptomatic, and behavioral factors. The findings underscore the need for targeted interventions, including awareness campaigns, preventive strategies, and streamlined healthcare access, to address the unique challenges that different subgroups of teachers face. Future research should further explore longitudinal trajectories and objective measures to enhance our understanding of VD in educational settings.

## Introduction

Voice disorders (VD) among teachers have become a subject of increasing concern, given educators' pivotal role in society and the potential impact of vocal impairments on their professional and personal lives [1-3]. The human voice is a complex and delicate instrument, and individuals who rely on their voices for extended periods, such as teachers, are particularly vulnerable to vocal health issues [1,2].

Teaching is a vocally demanding profession that requires clear articulation, projection, and sustained vocalization [1,4]. The classroom environment often necessitates elevated voice volume to capture students' attention and maintain order [5]. Additionally, teachers engage in various vocal tasks, including explaining concepts, managing discussions, and addressing diverse classroom situations. This constant and multifaceted use of the voice creates an occupational context where the risk of developing VD is heightened [4,6].

VD encompass a range of conditions affecting the vocal cords and surrounding structures, leading to alterations in pitch, volume, and quality of the voice [2]. Common manifestations include hoarseness, throat pain, and difficulty sustaining prolonged speech [7,8]. These conditions can be broadly classified into functional and organic disorders, with functional disorders arising from improper voice use and organic disorders involving structural abnormalities [1-3].

The prevalence of VD among teachers has been the subject of numerous studies, reflecting the significance of this occupational health concern [3-6]. Research suggests that teachers are at a greater risk of developing VD than individuals in other professions [2-5,6]. The impact of these disorders extends beyond the physical realm, affecting teachers' psychosocial well-being and professional efficacy. Persistent voice problems can lead to absenteeism, reduced job satisfaction, and even considerations of early retirement [6,7].

Several factors contribute to the heightened susceptibility of teachers to VD [5-7,9]. These include the need for frequent projection, exposure to noisy environments, and the performance of additional vocal tasks, such as singing or participating in school events [10,11]. Individual factors, such as age, gender, and personal health practices, may also influence the likelihood of developing voice problems [6,7,12].

Understanding how teachers respond to VD is crucial for developing targeted interventions and support systems [2,6]. Health-seeking behavior refers to the actions individuals take to address perceived health issues, and exploring this topic within the context of teachers and VD is a relatively underexplored area [1,2,9]. Factors influencing whether teachers seek medical advice, the timing of seeking help, and the reasons for not seeking advice are all essential aspects of comprehending and addressing this health concern [4,7].

The complex interplay of occupational demands, individual factors, and health-seeking behavior makes VD among teachers a multifaceted issue deserving of in-depth exploration.

This study aims to contribute to the existing body of knowledge by shedding light on the prevalence of VD among Taif City teachers and the factors that lead to those disorders. Additionally, knowing if those teachers are seeking health or not, we ultimately facilitate the development of strategies to promote vocal health within the educational setting and improve it.

Given the centrality of effective communication in the teaching profession, VD represent not only a personal challenge for educators but also a potential impediment to the quality of education. Investigating the health-seeking behavior of teachers regarding VD contributes to our understanding of the factors influencing the decision-making process. This knowledge can inform the development of targeted interventions, preventive measures, and support mechanisms to enhance vocal health and, by extension, the overall well-being of teachers.

## Materials and methods

Study design

This cross-sectional observational study was conducted in Taif City schools between November and December 2023. This study aimed to comprehensively explore the health-seeking behavior of teachers regarding VD. The study design allowed the collection of data at a single point in time, providing a snapshot of participants' characteristics and behaviors related to seeking medical advice for VD.

The study was conducted in various educational settings within Taif City, including educational institutions at different levels (kindergarten, primary, intermediate, and high school). These settings ensured the inclusion of teachers from different backgrounds and with different experiences, allowing for a comprehensive representation of the population.

A minimum of 311 teachers was determined as the required sample size to ensure reliable representation. This calculation was performed using Raosoft with a confidence level of 95% and a margin of error of 5%.

Inclusion and exclusion criteria

The participants eligible for this study were teachers aged 21 to 60 years, working in Taif for at least one year, and actively involved in teaching activities. Additionally, all participants provided informed consent before they could participate in the study. Teachers who did not complete one year of teaching, teachers who did not participate actively in teaching, and those who did not complete the survey were excluded from the study.

Data collection

The data were collected through a questionnaire the authors adopted from previous studies [13,14] designed to capture information on demographic variables, symptoms of VD, health-seeking behaviors, and voice handicap index-10 (VHI-10) scores. The questionnaire was distributed electronically to ensure broad participation and efficient data collection.

Participants were asked about demographic variables such as nationality, residency, age, gender, marital status, number of offspring, smoking status, and voice habits. These variables were chosen to provide a comprehensive understanding of the study population.

The occupational variables included years of experience as a teacher, educational level taught, teaching subject, number of students in the class, and the number of classes taught weekly. These variables aimed to capture the diverse aspects of teachers' professional backgrounds.

Teachers were asked about specific voice-related symptoms experienced during teaching, such as throat pain, sudden choking, a mass in the throat, the need to clear the throat, hoarseness, throat dryness, difficulty swallowing, difficulty breathing, difficulty talking, and associated conditions such as allergic rhinitis, bronchial asthma, recurrent upper respiratory infections, chronic cough, and reflux.

The participants were asked about the impact of VD on their lives. Participants were asked if they suffer from VD, frequently miss work, have considered resigning due to their condition, or if the voice symptoms limit their daily activity. The participants were questioned about their health-seeking behavior in response to VD, awareness of vocal training and hygiene, and methods to avoid VD.

The Arabic-validated version of the VHI-10 was utilized to distinguish between teachers with and without VD. The VHI-10 comprises ten items, and a 5-point Likert scale is used for each item. The score for each item ranges from 0 to 4, with a total score ranging from 0 to 40. A total score of 11 or higher was considered abnormal [15]. The VHI was also used in previous studies for the same purpose [13,14].

Before distributing the questionnaire, we conducted a pilot study of 25 teachers to assess its validity and reliability and to resolve obstacles.

Data analysis

The data were processed using SPSS version 26 (IBM Corp. Released 2019. IBM SPSS Statistics for Windows, Version 26.0. Armonk, NY: IBM Corp.). Qualitative data are presented as numbers and percentages. Relationships between categorical variables were assessed using chi-squared tests (χ2). Statistical significance was set at p<0.05.

Ethical considerations

Ethical approval was obtained from the Taif University Ethical Committee (approval number: 45-070) before data collection. Informed consent was secured from all participants, ensuring voluntary participation and the confidentiality of the responses.

## Results

Prevalence of VD and demographic characteristics

The study involved a total of 568 teachers, 351 (61.8%) of whom had VD. The participants exhibited a diverse age distribution, with 321 (56.5%) falling between the ages of 41 and 50. Female teachers constituted 362 (63.7%) of the participants. Furthermore, 514 (90.5%) participants were nonsmokers. The distribution of VD across different age groups revealed a statistically significant difference (p=0.031). Notably, teachers aged 31-40 years exhibited the highest prevalence of VD (67.2%, n=90). A gender-based analysis demonstrated a substantial association (p<0.001), with females showing a greater incidence of VD (69.9%, n=253) than males (47.6%, n=98). Marital status did not exhibit a significant relationship with VD. Further investigation revealed a substantial relationship between the habit of using a loud voice in teaching and VD (p<0.001), with those using a loud voice showing a greater prevalence of VD (67.5%, n=262). However, using a loud voice in other activities did not demonstrate a significant association with VD. Interestingly, there was no significant association between smoking and VD (Table 1).

**Table 1:** VD in relation to demographic characteristics and habits of teachers (n=568) The p-value is considered significant at p<0.05 level; χ2: chi-squared test; VD: voice disorders

Parameter		VD		Total N (%)	χ2	p-value
		No N (%)	Yes N (%)			
Age (years)	21-30	12 (57.1)	9 (42.9)	21 (3.7)	8.853	0.031
	31-40	44 (32.8)	90 (67.2)	134 (23.6)		
	41-50	117 (36.4)	204 (63.6)	321 (56.5)		
	51-60	44 (47.8)	48 (52.2)	92 (16.2)		
Gender	Female	109 (30.1)	253 (69.9)	362 (63.7)	27.696	<0.0001
	Male	108 (52.4)	98 (47.6)	206 (36.3)		
Marital status	Unmarried	39 (37.9)	64 (62.1)	103 (18.1)	0.0062	0.937
	Married	178 (38.3)	287 (61.7)	465 (81.9)		
Number of children	None	32 (44.4)	40 (55.6)	72 (12.7)	2.353	0.308
	1-3	78 (40)	117 (60)	195 (34.3)		
	>3	107 (35.5)	194 (64.5)	301 (53)		
Smoking status	Non-smoker	191 (37.2)	323 (62.8)	514 (90.5)	2.499	0.114
	Smoker	26 (48.1)	28 (51.9)	54 (9.5)		
Loud voice in teaching	No	91 (50.6)	89 (49.4)	180 (31.7)	17.027	<0.0001
	Yes	126 (32.5)	262 (67.5)	388 (68.3)		
Loud voice in other activities	No	171 (38.5)	273 (61.5)	444 (78.2)	0.082	0.774
	Yes	46 (37.1)	78 (62.9)	124 (21.8)		

Teaching-related characteristics

Years of teaching did not exhibit a significant association with VD. Similarly, the level of teaching (kindergarten, primary, intermediate, or high school) did not significantly influence the overall education level. However, the teaching subject demonstrated a noteworthy association (p=0.012), with English language teachers showing a higher prevalence of VD (77.3%, n=51). An analysis of the number of students in the class revealed a significant association with VD (p=0.001), where teachers of classes with more than 30 students exhibited a greater prevalence of VD. Additionally, the number of classes taught per week was significantly associated with VD (p<0.001), with teachers having more than 20 classes per week having a higher incidence of VD (Table 2).

**Table 2:** VD in relation to teaching-related characteristics of teachers (n=568) The p-value is considered significant at p<0.05 level; χ2: chi-squared test; VD: voice disorders

Parameter		VD		Total N (%)	χ2	p-value
		No N (%)	Yes N (%)			
Years of teaching	1-10	28 (36.4)	49 (63.6)	77 (13.6)	1.072	0.585
	10-20	86 (36.3)	151 (63.7)	237 (41.7)		
	>20	103 (40.6)	151 (59.4)	254 (44.7)		
Level	Kindergarten	9 (40.9)	13 (59.1)	22 (3.9)	2.919	0.404
	Primary school	94 (38.5)	150 (61.5)	244 (43)		
	Intermediate school	38 (31.9)	81 (68.1)	119 (21)		
	High school	76 (41.5)	107 (58.5)	183 (32.2)		
Main teaching subject	Primary and Kindergarten	21 (60)	14 (40)	35 (6.2)	19.569	0.012
	Social science	13 (40.6)	19 (59.4)	32 (5.6)		
	Art and physical	8 (28.6)	20 (71.4)	28 (4.7)		
	Information technology	11 (42.3)	15 (57.7)	26 (4.6)		
	Religion studies	33 (45.2)	40 (54.8)	73 (12.9)		
	Mathematics	26 (31)	58 (69)	84 (14.8)		
	English	15 (22.7)	51 (77.3)	66 (11.6)		
	Arabic	60 (42.3)	82 (57.7)	142 (25)		
	Natural sciences	30 (36.6)	52 (63.4)	82 (14.4)		
Number of students in the class	<20	38 (55.1)	31 (44.9)	69 (12.1)	13.096	0.001
	20-30	87 (40.7)	127 (59.3)	214 (37.7)		
	>30	92 (32.3)	193 (67.7)	285 (50.2)		
Number of classes per week	<10	53 (55.8)	42 (44.2)	95 (16.7)	19.716	<0.001
	10-20	140 (37.1)	237 (62.9)	377 (66.4)		
	>20	24 (25)	72 (75)	96 (16.9)		

Health-related characteristics

The prevalence of specific voice-related symptoms is prominently highlighted, revealing statistically significant associations. Hoarseness and throat pain were notably prevalent among teachers with VD (p<0.001). Similarly, other symptoms, such as choking, globus sensation, and throat dryness, were also significantly more prevalent in participants with VD (p<0.001). The association between VD and related health conditions is also evident. Allergic rhinitis, bronchial asthma, recurrent upper respiratory infections, chronic cough, and reflux were significantly related to VD (p<0.001). Notably, 81.4% (n=57) of the teachers with a family history of vocal problems also experienced VD (p<0.001) (Table 3).

**Table 3:** VD in relation to health-related characteristics of teachers (n=568) The p-value is considered significant at p<0.05 level; χ2: chi-squared test; VD: voice disorders

Parameter		VD		Total N (%)	χ2	p-value
		No N (%)	Yes N (%)			
Voice-related symptoms	Throat pain	49 (21.5)	179 (78.5)	228 (40.2)	384.707	<0.001
	Chocking	1 (2)	48 (98)	49 (8.6)		
	Globus sensation	12 (15.8)	64 (84.2)	76 (13.4)		
	Throat clearing	24 (13.6)	153 (86.4)	177 (31.2)		
	Hoarseness	41 (15.7)	220 (84.3)	261 (46)		
	Throat dryness	98 (30.9)	219 (69.1)	317 (55.9)		
	Difficulty swallowing	10 (18.5)	44 (81.5)	54 (9.5)		
	Difficulty breathing	6 (11.5)	46 (88.5)	52 (9.2)		
	Difficulty talking	5 (9.4)	48 (90.6)	53 (9.3)		
	None	37 (97.4)	1 (2.6)	38 (6.7)		
Associated health conditions	Allergic rhinitis	55 (24.8)	167 (75.2)	222 (39.1)	114.106	<0.001
	Bronchial asthma	6 (18.8)	26 (81.3)	32 (5.6)		
	Recurrent upper respiratory infections	14 (17.3)	67 (82.7)	81 (14.3)		
	Chronic cough	14 (21.5)	51 (78.5)	65 (11.4)		
	Reflux	17 (23.9)	54 (76.1)	71 (12.5)		
	None	132 (54.5)	110 (45.5)	242 (42.6)		
Family history of vocal disorders	No	204 (41)	294 (59)	498 (87.7)	13.035	<0.001
	Yes	13 (18.6)	57 (81.4)	70 (12.3)		

Occupational impact of VD and coping measures

**Figure 1:**
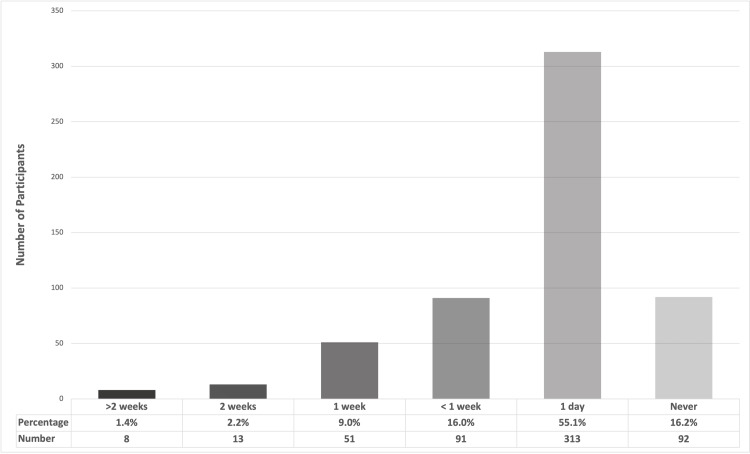
Duration of work absenteeism related to voice symptoms among participating teachers during the past year (n=568)

A substantial proportion of involved teachers (61.8%, n=351) reported suffering from voice symptoms related to teaching, highlighting the occupational burden of VD. Frequent work absenteeism due to voice problems was observed in 28.7% (n=163) of the teachers, signifying the tangible impact of voice problems on professional activities (Figure 1). Furthermore, a noteworthy percentage of teachers (6.3%, n=36) considered retirement or resignation due to voice problems, emphasizing the importance of these issues in career decisions. Teachers experiencing VD report limitations in daily or teaching activities (33.3%, n=189), emphasizing the pervasive impact on overall functioning. However, only 27.8% (n=158) of the teachers sought medical advice for their VD. In comparison, 26.8% (n=152) were aware of the importance of vocal training and vocal hygiene, suggesting a potential gap in awareness and knowledge (Table 4).

**Table 4:** Impact of VD on teachers and their attitude and awareness (n=568) VD: voice disorders

Parameter	N (%)
Frequent work absenteeism because of voice problems	163 (28.7)
Considered retirement or resignation due to voice problems	36 (6.3)
Voice symptoms limit daily or teaching activities	189 (33.3)
Seeking medical advice regarding VD	158 (27.8)
Aware of the importance of vocal training and vocal hygiene	152 (26.8)
Interesting in attending workshops to increase awareness about VD	392 (69)

**Figure 2:**
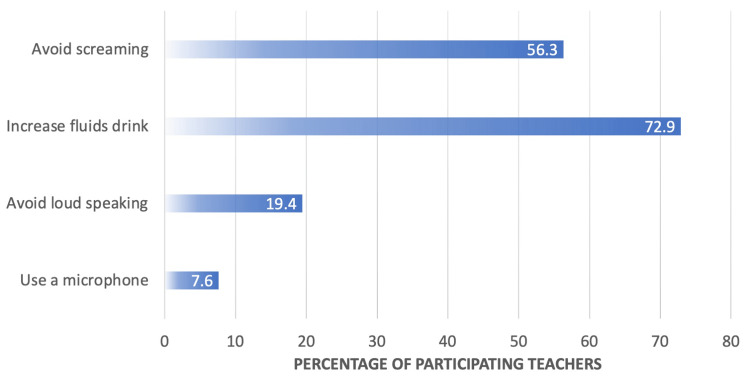
Methods used by the teacher to avoid vocal problems during teaching (n=568)

Notably, the majority of teachers (72.9%, n=414) increase fluid intake as a preventive measure, while avoiding screaming is also a prevalent strategy (56.3%, n=320). Using a microphone (7.6%, n=43) and avoiding loud speaking (19.4%, n=110) are less commonly reported methods. These findings illuminate the diverse strategies teachers employ to mitigate the risk of vocal problems, emphasizing the importance of incorporating these insights into educational and preventive interventions (Figure 2).

## Discussion

VD among teachers poses a significant occupational health concern, given the fundamental role of effective communication in teaching [1,3]. The unique demands placed on teachers' vocal apparatus during sustained speech and projection in the classroom make them particularly susceptible to voice-related issues [5,6]. Understanding the prevalence, associated factors, and impact of VD among teachers is crucial for developing targeted interventions and support systems [7-9]. This study aimed to thoroughly examine the prevalence and impact of VD on teachers in Taif, KSA.

The findings of our study involving 568 teachers reveal a nuanced landscape of VD among educators. Our research revealed that 61.8% of teachers suffer from VD, highlighting the significant impact of this occupational hazard. Throat pain, hoarseness, and throat dryness were the most commonly reported symptoms. These findings align with previous research indicating that teaching is particularly susceptible to VD due to prolonged and often strenuous vocal use in the classroom [2,6,11].

Notably, age, gender, and occupational factors such as teaching subject, class size, and weekly teaching load are associated with the prevalence of VD. Furthermore, some related conditions, such as reflux and allergic rhinitis, demonstrate significant correlations with VD [5,6]. The impact of VD on teachers' professional and personal lives is evident, with a substantial percentage reporting symptoms related to teaching, work absenteeism, and even considering retirement due to voice problems [7,8].

Among the notable findings, the association between age and VD is particularly intriguing. Teachers aged 31-40 years exhibited the highest prevalence of VD. These findings align with those of several previous studies highlighting an increased susceptibility to vocal issues during the mid-career phase, possibly attributed to a combination of factors such as prolonged exposure to teaching environments and evolving lifestyle habits [16-18]. Conversely, teachers aged 51-60 also showed a notable prevalence of bias, suggesting that vocal health challenges may persist or emerge later in a teacher's career. These findings emphasize the importance of implementing preventative measures and tailored interventions targeting teachers in various career stages [19].

The gender-based disparity in the incidence of VD is another crucial observation. There is a significantly greater prevalence of female teachers than male teachers [5,8]. This finding aligns with the literature, which often attributes the increased vulnerability of females to biological differences, hormonal fluctuations, and societal expectations regarding vocal intensity and pitch [2,6,20]. Addressing these gender-specific factors in vocal health education and support initiatives is imperative to ensure equitable care and prevention strategies for all teachers.

Upon closer examination, our study revealed no significant difference in the incidence of VD between married and unmarried teachers. These findings align with previous assumptions and suggest that marital status may not substantially impact the development of VD [6].

Occupational variables, including teaching subject, class size, and weekly teaching load, also significantly correlated with VD. Teachers teaching larger classes and more weekly hours face a greater risk of VD. This finding aligns with the literature emphasizing the impact of increased vocal demand on teachers, especially in crowded classrooms or when teaching subjects requires heightened vocal projection [5,6,8].

Hoarseness, throat pain, and throat dryness were identified as key symptoms significantly associated with VD [2,10,19]. These prevalent symptoms are consistent with the occupational strain experienced by teachers [12]. The association between allergic rhinitis and bronchial asthma further underscores the interconnectedness of respiratory health and vocal well-being [6]. Similar findings have been reported in studies emphasizing the impact of allergic conditions on vocal health, potentially through mechanisms such as increased mucus production or irritation of the vocal folds [4]. Strategies to manage allergic conditions and alleviate associated symptoms should be integrated into vocal health interventions for teachers.

Family history emerges as a significant factor, with teachers having a family history of vocal disorders exhibiting a greater prevalence of VD. This finding underscores the potential genetic component of vocal health, suggesting a hereditary predisposition. While limited studies have explored the heritability of VD, these findings open avenues for genetic research and understanding the interplay between genetic factors and environmental influences on vocal health [3,21].

Our findings align with and extend the literature on VD among teachers. Age-related trends are consistent with studies emphasizing the susceptibility of mid-career teachers, but our study sheds light on the persistence of these issues in later career stages. Gender-based disparities have been reported previously, and our study underscores the need for gender-sensitive interventions [6,11,17]. The impact of teaching variables, such as subject, class size, and weekly load, on vocal health, is supported by the literature, emphasizing the need for tailored strategies for specific teaching contexts [6].

The correlation between respiratory conditions and VD has been supported by studies linking respiratory health to vocal health [6,20,21]. However, the aspect of family history introduces a novel dimension, suggesting a potential genetic influence. While limited research has delved into the genetic factors of VD, our study prompts further exploration into familial patterns and their implications for preventive measures.

Study strengths and limitations

This study has several strengths that enhance its robustness and relevance. The inclusion of a diverse and sizable sample of 568 teachers from various educational settings ensures a comprehensive representation of the population, contributing to the external validity of the study. The utilization of a validated questionnaire adapted from previous studies enhances the reliability of the collected data, and the electronic distribution method facilitates broad participation and efficient data collection. The incorporation of demographic, occupational, and health-related variables provides a holistic understanding of the factors associated with VD among teachers. Moreover, the rigorous pilot study conducted with 25 teachers ensures the validity and reliability of the questionnaire, contributing to the methodological soundness of the research.

Despite its strengths, this study has certain limitations. The cross-sectional design precludes the establishment of causal relationships, and reliance on self-reported data introduces the potential for recall bias and subjective interpretation. While the sample is representative of Taif City teachers, generalizability to other regions may be limited. The exclusive use of a questionnaire may oversimplify the multifaceted nature of VD, and the focus on health-seeking behavior may not capture the full spectrum of coping strategies employed by teachers. The absence of a control group restricts the ability to compare prevalence rates with those of nonteaching populations. Despite these limitations, the study's comprehensive approach provides valuable insights into the prevalence, associated factors, and impact of VD among teachers, laying a foundation for future research and targeted interventions.

## Conclusions

Our study comprehensively examined VD among teachers in Taif. The findings offer insights into demographic, occupational, and health-related factors influencing the prevalence and impact of VD. The identification of age, gender, and specific symptoms as significant factors informs the development of targeted interventions for at-risk teacher populations. Additionally, the association with family history highlights potential genetic influences, suggesting avenues for further research. Understanding the multifaceted nature of VD among teachers is crucial for implementing effective preventive measures and support systems. The implications of our findings extend beyond individual health, impacting teaching quality, professional longevity, and overall occupational well-being. By incorporating these insights into educational and occupational health initiatives, we can foster a more supportive environment for teachers, ensuring the preservation of their vocal health and, consequently, the enhancement of their overall professional experience.
